# Agonistic Autoantibodies to the β2-Adrenergic Receptor Involved in the Pathogenesis of Open-Angle Glaucoma

**DOI:** 10.3389/fimmu.2018.00145

**Published:** 2018-02-12

**Authors:** Anselm Jünemann, Bettina Hohberger, Jürgen Rech, Ahmed Sheriff, Qin Fu, Ursula Schlötzer-Schrehardt, Reinhard Edmund Voll, Sabine Bartel, Hubert Kalbacher, Johan Hoebeke, Robert Rejdak, Folkert Horn, Gerd Wallukat, Rudolf Kunze, Martin Herrmann

**Affiliations:** ^1^Department of Ophthalmology, University of Rostock, Rostock, Germany; ^2^Department of Ophthalmology, Friedrich-Alexander-University of Erlangen-Nürnberg, Erlangen, Germany; ^3^Department of Internal Medicine III, Institute of Clinical Immunology and Rheumatology, University of Erlangen-Nürnberg, Erlangen, Germany; ^4^Department of Pharmacology, Tongji Medical College, Huazhong University of Science and Technology, Wuhan, China; ^5^Max Delbrück Center for Molecular Medicine, Berlin, Germany; ^6^IZKF Research Group 2, Nikolaus-Fiebiger-Center of Molecular Medicine, University of Erlangen-Nürnberg, Erlangen, Germany; ^7^IFIB - Institute of Biochemistry, University of Tübingen, Tübingen, Germany; ^8^C.N.R.S. UPR 9021 «Chimie et Immunologie Thérapeutiques», Strasbourg, France; ^9^Department of General Ophthalmology, Medical University of Lublin, Lublin, Poland; ^10^Science Office, Berlin-Buch, Campus Max Delbrück Center for Molecular Medicine, Berlin, Germany

**Keywords:** autoantibodies, glaucoma, ocular hypertension, β2-adrenergic receptor, agonistic, immunoadsorption

## Abstract

Glaucoma is a frequent ocular disease that may lead to blindness. Primary open-angle glaucoma (POAG) and ocular hypertension (OHT) are common diseases with increased intraocular pressure (IOP), which are mainly responsible for these disorders. Their pathogenesis is widely unknown. We screened the sera of patients with POAG and OHT for the prevalence of autoantibodies (AAb) against G protein-coupled receptors (GPCRs) in comparison to controls. Employing frequency modulation of spontaneously contracting neonatal rat cardiomyocytes *in vitro*, agonistic GPCR AAb were to be detected in roughly 75% of the patients with POAG and OHT, however, not in controls. Using inhibitory peptides the AAb’ target was identified as β2 adrenergic receptor (β2AR). The AAb interact with the second extracellular loop of β2AR. The peptides 181–187 and 186–192 were identified as binding sites of the AAb within the extracellular loop II. The binding of the AAb to β2ARs was verified by surface-plasmon-resonance analysis. The isotype of the AAb was (immunoglobulin) IgG3. In an additional pilot principal-of-proof study, including four patients with POAG, the removal of the AAb against the β2AR and other immunoglobulins G by immunoadsorption resulted in a transient reduction of IOP. These findings might indicate a possible role of agonistic AAb directed against β2ARs in the dynamics of aqueous humor and might support a contribution of adaptive autoimmunity in the etiopathogenesis of POAG and OHT.

## Introduction

Glaucoma is one of the leading causes of blindness in the world. Over 67 million people are affected by glaucoma (1, 2). Elevated intraocular pressure (IOP) is the major risk factor for glaucoma. Primary open-angle glaucoma (POAG) is now defined as a progressive disease of retinal ganglion cells characterized by structural change in the optic disk and by typical, slowly progressive loss of function. The most common form of the group of glaucomatous diseases is POAG with about 80%. Ocular hypertension (OHT) is characterized by elevated IOP without optic nerve degeneration. One to 2% of patients with OHT per year convert to POAG.

The pathogenesis of the optic nerve damage in POAG is complex and associated with increased IOP, neurotoxicity and apoptosis (3, 4), changes of the extracellular matrix (5, 6), activation of glia cells (7), an interrupted transport of neutrophins (8), oxidative stress (9, 10), and hypoxia due to ocular and systemic vascular dysregulation (9). There are also reports of glaucoma-associated degenerative processes of the central visual pathways in the brain (11). Thus, new targets for therapeutic intervention, such as improving ocular blood flow and direct neuroprotection of retinal ganglion cells, are under investigation.

Since elevated IOP is the primary risk factor for the development and progression of glaucoma, the lowering of the IOP is the primary goal of all treatment strategies (12). Many studies have shown that IOP reduction can slow the progression of glaucoma (13–17) and delay or even prevent the onset of retinal ganglion cell loss. Lowering the IOP by 1 mmHg prevents progressive visual field loss by 10% (18). Conservative [e.g., β2-adrenergic receptor (β2AR) blocker] and surgical therapeutic options are offered to achieve IOP lowering. The β2AR blocker Timolol reduces the IOP in humans (19) by suppressing the rate of aqueous humor formation (20, 21) by 30–50% (20–26). The relative potency of the β2-adrenergic blocker Timolol is higher than that of the β1-adrenergic receptor blocker Betaxolol (27), indicating the prominent role of β2ARs in the formation of aqueous humor and/or regulation of its flow.

Trabecular meshwork and ciliary body express β2ARs (28, 29), regulating the aqueous humor system in the eye and consequently influencing IOP. Thus, β2AR plays a key role in the regulation of both aqueous humor production and outflow. In addition, β2AR is also expressed in human optic nerve and in microvessels (30, 31). β2ARs are members of the G protein-coupled receptors (GPCR) family, comprised of more than 600 genes (32, 33). They have seven membrane spanning domains, three intra- and extracellular loops, an extracellular N-terminus, and an intracellular C-terminal tail. Most of the GPCR form homodimers upon ligand activation. It has been shown that functional autoantibodies (AAbs), directed against GPCR, are associated with various human diseases. The first AAb against a GPCR was described for the β2AR in patients with allergic asthma in 1980 (34). Several groups identified agonistic AAb against the β1-adrenergic receptor in Chagas’ disease (35, 36), dilated cardiomyopathy (37–39), ischemic cardiomyopathy (40), and myocarditis (41). β2- and α1-AAb were seen in Alzheimer’s and vascular dementia (42). β1-, β2-, and α1-AAb were shown to be increased in patients with preeclampsia (43). α1-AAb were also detected in primary and malignant hypertension (44).

We hypothesize that AAb against GPCR might be involved in the pathogenesis of glaucomatous disease by influencing the dynamics of aqueous humor.

To study the hypothesis that circulating immunoglobulins of patients with glaucoma activate GPCRs, isolated rat cardiomyocytes were employed as target cell. Frequency modulation of spontaneously beating neonatal cardiomyocytes is highly efficient to investigate such targets (1–3, 37, 45). The rat cardiomyocytes contain a spectrum of relevant GPCRs, e.g., β1-, β2-, α-ARs, and Angiotensin-1 receptor, which display a high homology to their corresponding human receptors. The homology of the amino acids between the human and the rat second extracellular loop is 88%; however, the homology in the estimated binding site of the AAbs in this extracellular loop is 100%. The cells were exposed to IgG prepared from controls or patients with glaucoma and OHT. To verify the specificity of the effects, the heart cells were treated with receptor-specific agonists in the presence or absence of receptor-specific antagonists and compared with the AAb-mediated responses. In screening studies using respective agonists and blocking agents an interaction of the glaucoma-associated IgG with the β1AR, α-AR, or with AT1-receptor system has been excluded (not shown). Based on these findings, the potential importance of β2AR-directed antibodies associated with glaucoma was analyzed in more detail. These findings were the basis for the following experiments. Investigations of the target loop as well as the target epitope of the GPCR and the IgG isotype of the AAb were added. Surface plasmon resonance analysis confirmed the receptor specificity of the agonistic β2AR AAb.

In order to draw a bow between the molecular findings and clinical data, correlation analyses of the patients’ clinical parameter with the β2AR AAb were done. Finally, a pilot proof-of-principal study was performed in four glaucoma patients using an extracorporal immunoadsorption (IA) for removal of AABs against β2AR and other immunoglobulins G in order to monitor a potentially transient or permanent change of IOP.

## Materials and Methods

### Patients

Patients with POAG and patients with OHT were recruited from the Department of Ophthalmology of the University of Erlangen-Nürnberg (Friedrich-Alexander-Universität Erlangen-Nürnberg (FAU)). The control groups were from the Max Delbrück Centre of Molecular Medicine Berlin (MDC) and from the University of Erlangen-Nürnberg [normal healthy donors (NHD) and cataract patients].

All controls and patients were thoroughly examined by slit lamp inspection, applanation tonometry, funduscopy, gonioscopy, perimetry, and papillometry. In addition, a 24 h IOP curve was measured in all glaucoma patients (6 determinations: 7:00 a.m., 12:00 a.m., 5:00 p.m., 9:00 p.m., 12:00 p.m., and 7:00 a.m.). Papillometric evaluations of the patients were based on 15-color photographs and subsequent planimetry (Zeiss Morphomat 30) of the area of the optic disk and the neuroretinal rim area (46). Criteria for all glaucoma diagnoses were an open anterior chamber angle and glaucomatous changes of the optic nerve head including an unusually small neuroretinal rim area in relation to the optic disk size and higher vertical cup-to-disk ratios than horizontal ratios (45). All subjects underwent visual field testing with standard white-on-white perimetry using a computerized static projection perimeter (Octopus 500, Interzeag, Schlieren, Switzerland; program G1, 3 phases). Subjects with more than 12% false-positive or false-negative responses were not included in this study. A glaucomatous visual field was defined as an white-on-white visual field with (a) at least three adjacent test points having a deviation of equal to or greater than 5 dB and with one test point with a deviation greater than 10 dB lower than normal, (b) at least two adjacent test points with a deviation equal to or greater than 10 dB, (c) at least three adjacent test points with a deviation equal to or greater than 5 dB abutting the nasal horizontal meridian, or (d) a mean visual field defect of more than 2.6 dB. Exclusion criteria were all eye diseases other than glaucoma, OHT or cataract, respectively. The clinical characteristics of controls (cataract and NHD) and patients can be found in Tables 1 and 2. For the patients, antiglaucomatous therapy and general diseases are given in Tables 3 and 4.

**Table 1:** Clinical characteristics of patients with primary open-angle glaucoma (POAG) and ocular hypertension (OHT).

	POAG	OHT
Number	39	9
Age (years)	71.18 ± 8.8 (48–86)	51.0 ± 7.7 (42–68)
Gender	12 m (31%), 17 f (69%)	5 m (56%), 4 f (44%)
Glaucoma history (years)	15.2 ± 11.1 (1–39)	6.8 ± 3.9 (3–14)
IOPmax (mmHg) OD	34,2 ± 12,7 (20–67)	28.7 ± 3.7 (23–35)
IOPmax (mmHg) OS	33.0 ± 12.2 (19–67)	26.9 ± 4.1 (22–35)
IOPact (mmHg) OD	17.8 ± 6.8 (10–38)	15.7 ± 3.9 (9–21)
IOPact (mmHg) OS	17.5 ± 5.7 (9–42)	17.4 ± 3.7 (13–22)
MD (dB) OD	10.8 ± 7.9 (−4.3 to 25.0)	−0.13 ± 0.96 (−1.5 to 1.5)
MD (dB) OS	11.9 ± 7.9 (−0.7 to 28.6)	0.25 ± 1.1 (−1.4 to 1.8)
CLV (dB^2^) OD	42.2 ± 32.1 (4.0–112.7)	3.15 ± 1.42 (1.5–5.8)
CLV (dB^2^) OS	40.7 ± 26.1 (1.7–124.0)	3.1 ± 1.7 (1.8–6.8)
Visual acuity OD	0.59 ± 0.3 (0.002–1.2)	1.0 ± 0.1 (1.0–1.2)
Visual acuity OS	0.59 ± 0.3 (0.004–1.0)	1.1 ± 0.1 (0.8–1.2)

*Values are given as mean ± SD (range)*.

*IOPmax, maximal intraocular pressure; IOPact, actual intraocular pressure; MD, mean defect; CLV, corrected loss variance; OD, right eye; OS, left eye; m, male; f, female*.

**Table 2:** Clinical characteristics of control group.

	Cataract patients	Normal healthy donors
Number	8	9
Age (years)	71.0 ± 10.7 (55–83)	44.6 ± 15.4 (22–65)
Gender	3 m (37%), 5 f (63%)	1 m (11%), 8 f (89%)
IOPact (mmHg) OD	13.3 ± 2.5 (10–17)	
IOPact (mmHg) OS	13.6 ± 3.4 (10–21)	
Visual acuity OD	0.47 ± 0.3 (0.002–1.0)	
Visual acuity OS	0.59 ± 0.3 (0.002–1.0)	

*IOPact, actual intraocular pressure; OD = right eye. OS, left eye; m, male, f, female*.

**Table 3:** Glaucoma therapy of patients with primary open-angle glaucoma (POAG) and ocular hypertension (OHT).

Therapy	POAG (*n*)	OHT (*n*)
Antiglaucomatous medication	10	7
Glaucoma surgery without medication	6	0
Glaucoma surgery with medication	23	0
Number of antiglaucomatous medication		
0	3	2
1	13	4
2	5	3
3	11	0
4	4	0
Antiglaucomatous medication		
Betablockers	23	1
Prostaglandins	21	7
CAI	19	1
A2-agonists	7	1
Pilocarpine	4	0

*CAI, carboanhydrase inhibitor*.

**Table 4:** General diseases of patients with primary open-angle glaucoma (POAG) and ocular hypertension (OHT).

	POAG	OHT
Diabetes	9 (23%)	0
Arterial hypertension	20 (51%)	3
Heart insufficiency	2 (5%)	0
Coronary heart disease	5 (13%)	0
Myocardial infarction	2 (5%)	0
Cardiac arrhythmia	3 (8%)	0
Stroke	3 (8%)	0
Immunological disease	4 (10%)	0
Rheumatoid arthritis	1	
Muscle rheumatism	1	
Psoriasis	1	
Raynaud’s phenomenon	1	
Thyroid disease	7 (18%)	0

The study followed the tenets of the declaration of Helsinki for research. Written informed consent to use serum samples for research purposes was obtained from each participants of the study. The institutional review board of the University Hospital Erlangen approved the protocols.

#### POAG Patients

The patients of the glaucoma group were referred by ophthalmologists for further diagnosis and follow-up of glaucoma. The POAG group included 39 patients with POAG, characterized by IOP measurements higher than 21 mmHg. All POAG patients had glaucomatous optic disk damage and pathological cumulative perimetric defect curves, i.e., local and/or diffuse visual field loss in white-on-white perimetry.

#### OHT Patients

The OHT group included 9 patients with elevated IOP (24 mmHg at least in one eye) showing normal optic disk and normal retinal nerve fiber layer. Computerized visual field examinations with white-on-white perimetry (Octopus program G1) were normal.

#### Control Group

This group included eight patients referred by ophthalmologists for cataract surgery. Slit-lamp inspection, tonometry, fundoscopy, and papillometry were normal in control subjects. In addition, nine NHD were included.

### Methods

#### Cardiomyocyte Bioassay—Isolation and Culture of Cardiac Myocytes

Primary cultures of cardiac myocytes were prepared from ventricle of 1- to 2-day-old Sprague-Dawley rats as described previously (37). Briefly, the myocardial cells were dispersed by digestion with a 0.25% solution of crude porcine trypsin (Serva, Germany) and were suspended in a SM20-I medium (Biochrom, Germany), containing 10% heat-inactivated neonatal calf serum (Gibco, Germany), streptomycin (HEFA Pharma; Germany), penicillin (Heyl, Germany), hydrocortisone (Merck, Germany), glutamine (Serva, Germany), and fluorodeoxyuridine (Serva, Germany), the latter to prevent proliferation of non-muscle cells. The cardiomyocytes were plated at a field density of 160,000 cells/cm^2^. Twenty-four hours after seeding, the culture medium was renewed and the cells were cultured for 3–4 days at 37°C before stimulation. The medium was replaced with fresh culture solution 2 h before being used in experiments. Seven to 10 selected cells or synchronously contracting cell clusters per flask were counted for 15 s on a heated stage of an inverted microscope at 37°C. The basal contraction rate of the spontaneously beating cardiomyocytes was measured to be 162 ± 4 min under these conditions. If not noted otherwise, the cardiomyocytes were incubated with immunoglobulin fractions derived from sera of healthy persons and patients for 60 min in a dilution of 1:40. The immunoglobulins were added in excess (in 50 μl: 0.4–0.5 mg IgG fraction; this volume was added to 2 ml culture medium/flask). The agonistic and antagonistic drugs for the β2AR, peptides, etc. were added to the heart cells singly or cumulatively as indicated. β2AR activation was induced by the β2AR agonist clenbuterol (Sigma, Germany, concentration of 1 μM) and inhibited by the specific β2AR blocker ICI118.551 (Sigma, Germany, concentrations of 0.1–0.3 μM) applied 5 min before. Then, IgG-containing fractions of the sera from glaucoma patients were added to cardiomyocytes in a standard dilution. The effect of the IgG fractions was antagonized by ICI118.551. Performance and in-house validation and reproducibility of the bioassay were tested previously (47).

#### Preparation of the Serum Immunoglobulin Fractions

The immunoglobulin fractions were prepared from sera by direct ammonium sulfate precipitation (final concentration of 40%). After overnight incubation at 4°C the precipitates were centrifuged at 4,000 × *g* for 30 min, and the pellets were dissolved in dialysis buffer (154 mmol/l NaCl, 10 mmol/l Na_2_HPO_4_/NaH_2_PO_4_, pH 7.2). The procedure of precipitating (50% final concentration of ammonium sulfate for a removal of small biological active compounds such as catecholamines or peptides), washing, and dissolving was repeated twice. Prior to the assays all samples were extensively dialyzed against phosphate-buffered saline at 4°C for 3 days. The buffer was changed twice daily. After ammonium sulfate precipitation a concentration of 800–900 mg protein per milliliter was measured. Fifty microliters of this protein amount were inserted for analyses. The immunoglobulin fractions were stored at −20°C until use.

### Affinity Purification of AAb

IgG preparations of patients with POAG and OHT were affinity purified using the biotinylated peptide biotin-AINCYANETCCD corresponding to the second extracellular loop of the β2AR. One milliliter of IgG was treated with 300 μl of the peptide (100 μg/ml) for 1 h. The peptide–antibody complex was incubated with streptavidin-coated magnetic particles (Roche, Germany) for 30 min. The separation was performed with a magnetic separator (Dynal, Germany). The particles were washed five times with PBS. The antibodies were eluted with 3 M KSCN in two 0.5 ml steps. The antibody solution was dialyzed against PBS for 48 h at 4°C.

#### Identification of the Target Loop of AAb against β2AR

β2AR-derived peptides identical to the extracellular loops I, II, or III were employed to identify the loop that interacts with the immunoglobulins isolated from the sera of patients with glaucoma. The following peptides were selected:

loop I (HILMKMWTFGNFWCEFWT)
loop II (HWYRATHQEAINCYANETCCDFFTNQ)
loop III (VIQDNLIRKEV).

The synthetic loop peptides were added in excess (0.5 μg in 50 μl) to 50 μl of the immunoglobulin fraction. The mixtures were shaken and incubated at room temperature for 1 h. The samples were then added to neonatal rat cardiomyocytes cultured in 2 ml of medium to a final IgG dilution of 1:40. Sixty minutes after addition of the peptide/immunoglobulin mixture the beating rate was monitored for 15 s.

#### Epitope Screening on Extracellular Loop II of β2AR

To identify the epitopes of the second extracellular loop of β2AR, mapping studies with small overlapping synthetic peptides were performed. The interacting sites between specific regions within the second extracellular loop and the IgG from glaucoma patients were screened with the following peptides:

HWYRAT (AS172–177),
ATHQEAI (AS176–182),
AINCYAN (AS181–187),
ANETCCD (AS186–192),
DFFTNQ (AS192–197).

The epitope analysis was performed similar as the loop screening.

#### IgG Subclass Analysis of the AAb against β2AR

To determine the IgG subclass of glaucoma-associated AAb against β2AR, IgGs from selected patients were treated with murine monoclonal anti-human IgG1, 2, 3, and 4 antibodies (SEROTEC, Germany). According to previous experiments (data not shown here), 3 μl of these antibodies were added to 50 μl of the IgG preparations. After 1 h at RT the samples were treated with 3 μl of a polyclonal anti-mouse Fc antibodies for 1 h to increase the complex. The samples were centrifuged at 10,000 × *g* and the supernatants were used in the cardiomyocyte bioassay at a dilution of 1:40.

#### Surface Plasmon Resonance

Binding of the AAb to the β2AR fragments was verified and quantified by surface-plasmon-resonance analysis (BIAcore). The IgG fractions of patients or controls were passed over biotinylated peptides loaded on a streptavidin-biosensor (BIAcore3000) at a flow rate of 5 μl/min. As β2AR-derived loop II peptide H19C (biotinyl-LC-HWYRATHQEAINCYANETC) was used. A biotinylated unrelated peptide served as control and its sensorgrams were subtracted from the specific signals. The initial linear association phase slope under conditions of high density of ligand is directly correlated with the molar concentration of the binding molecules.

### Pilot Proof-of-Principal Study on IA for the Treatment of Patients with POAG

In a pilot proof-of-principal study IA was performed in four patients with POAG to prospectively study safety and pressure-reducing efficacy of IA by unspecific removal of IgG and thereby the reduction of agonistic AAbs. The study followed the tenets of the declaration of Helsinki for research and was approved by the local Ethics Committee (3483; ClinicalTrials.gov Identifier NCT00494923). All subjects signed an informed consent form before participating in any part of the study. Data of a follow-up of at least half a year are presented. Levels of AAb and immunoglobulins as well as IOP were monitored before IA (visit 0: with local antiglaucomatous eye drops; visit 1: with systemic antiglaucomatous medication, yet without local antiglaucomatous eye drops), during IA as well as randomly 2 weeks, 2, 4, and 6 months after IA. At each visit, the IOP was measured six times a day (7:00 a.m., 12:00 p.m., 5:00 p.m., 9:00 p.m., 12:00 a.m., 7:00 a.m. of the following day). Mean IOP was calculated for each patient for statistical analysis.

#### Patients

The clinical characteristics of the patients are displayed in Table 5. All four patients suffered from POAG and were on maximal combination therapy with antiglaucomatous eye drops without any surgical procedure in the past. Only in the first patient, the left eye was excluded from the statistical analysis since he had undergone trabeculectomy in the past. Inclusion criteria were the failure of the current therapy to control IOP and the refusal of surgical procedures. Two female and two male Caucasian patients aged between 52 and 71 years were included. Systemic diseases of the patients were:

patient 1 nephrectomy because of cancer in 1995,
patient 2 arterial hypertension,
patient 3 asthma bronchiale, hyperthyreosis,
patient 4 spine arthrosis, cyste of the hip, tinnitus.

**Table 5:** Pilot proof-of-principal study: demographic data of the four glaucoma patients.

Patient	Age (years)	Race	Sex	Diagnosis	Perimetric mean defect before IA (dB)		Glaucoma history (years)	*R*× before	*R*× in the end
					RA	LA			
1	71	Caucasian	M	OAG	11.7	17.0	12	Dorzolamid 2×	none
								Timolol 0.5% 2×	
								Travoprost 1×	

2	52	Caucasian	F	OAG	−0.9	0.0	6	Brimonidin 2×	Dorzolamid 2×
									Timolol 0.5% 2×
								Timolol 0.5% 2×	Latanoprost 1×
								Latanoprost 2×	

3	56	Caucasian	F	OAG	6.8	8.0	5	Dorzolamid 2×	Dorzolamid 1×
								Timolol 0.5% 2×	Timolol 0.5% 1×

4	57	Caucasian	M	OAG	−2.8	0.1	9	Clonidin 4×	none
								Dorzolamid 2×	
								Timolol 0.5% 2×	
								Latanoprost 1×	

#### Immunoadsorption

All four patients underwent one treatment cycle of five consecutive days from Monday to Friday. Two Globaffin^®^ adsorbers (double-columns system, columns for re-use; Fresenius Medical Care Affina GmbH) were used (48). Each adsorber contains 60 ml of a matrix based on sepharose CL-4B, suspended in sodium citrate buffer (10 mM; pH 4.0). One adsorber contains at least 250 mg of PGAM146 peptide covalently bound to Sepharose. The full synthetic peptide PGAM146 is a ligand for IgGs and binds them independent of the antigen specificity. The synthetic peptide PGAM-146 mimics a protein A ligand. In Globaffin the peptide is used as ligand to bind immunoglobulins from human plasma. The high affinity to all immunoglobulins (esp. IgG) or immunocomplexes that contain this Fc-part makes the peptide to a potential ligand for the removal of these substances. The peptide ligand is immobilized to cross linked agarose (Sepharose CL-4B). During treatment, the columns were regenerated with special buffer solutions (15 g/l glycine-hydrochloric acid, pH 2.8 and 8 g/l NaCl, 0.2 g/l KCL, 3.6 g/l Na_2_HPO_4_ × 12 H_2_O (phosphate-buffered saline, PBS) solution). After termination of the treatment, the Globaffin^®^ adsorbers were preserved using bacteriostatic and toxic buffers [0.4 g/l polyhexamethylene-biguanide (PHMB), 0.3 g/l tryptophan, 3.3 g/l sodium citrate dihydrate, 5.4 g/l sodium acetate trihydrate, 2.9 g/l sodium monohydrogen phosphate × 12 H_2_0, 0.26 g/l potassium dihydrogen phosphate, pH 7.0 (PHMB-Trp) solution]. For preservation, each adsorber was rinsed with at least 350 ml of preservation buffer [0.1 g/l sodium azide (PBS, 0.1%)]. Afterward, the adsorbers were stored at 2–10°C until the next treatment cycle. Extracorporal plasma separation was performed by centrifugation (Cobe Spectra^®^) while the running parameters, such as column volume, loading volume (250 ml/cycle), desorption and regeneration volume, speed of processing, and numbers of cycles, were fixed and controlled by the Adasorb^®^ device (medicap, Ulrichstein). Prior to connecting the patient to the system, the adsorber was washed with 2 l of 0.9% NaCl solution. During treatment, the plasma was alternately passed through the adsorbers. One adsorber served for the removal of immunoglobulins, the second was simultaneously regenerated by a glycin buffer. The amount of plasma to be treated (plasma volume, PV) was calculated before the treatment [females: PV (ml) = *a* × 9.03 + *b* × 24.13 − 766; males: PV (ml) = *a* × 19.9 + *b* × 13.1 − 2,000 (*a* = body height in cm; *b* = body weight in kg)].

#### Procedure of IA

Before the procedure, a 1250 IE heparin i.v. bolus was given. Access to circulation was provided *via* the cubital vein. Peripheral blood was drawn, anti-coagulated, and separated into plasma and blood cells. Extracorporal anti-coagulation was achieved by sodium citrate (ACD-A, 2.2%; 1:15–1:20 v/v to blood). The plasma was pumped through an adsorber column (Globaffin^®^) regulated by the Adasorb^®^ device. The separated plasma was perfused through the columns at a flow rate between 11 and 28 ml/min; one cycle contained 250 ml plasma. The treated plasma was subsequently remixed with the other blood components and reinfused. During one cycle of treatment, the twofold volume of the individually calculated plasma volume was cleared by the adsorber.

### Statistical Analysis

Student’s *t*-test was used to compare variables between the groups. Values of *P* < 0.05 are significant. Continuous data are presented as medians (with ranges). We performed comparisons between groups with use of Fisher’s exact test for categorical values and the Mann–Whitney *U* test for continuous variables. In addition, statistical analysis was done using four-field analysis.

## Results

### Sera of Patients with POAG and OHT Contain AAb Stimulating Rat Cardiomyocytes

**Figure 1:**
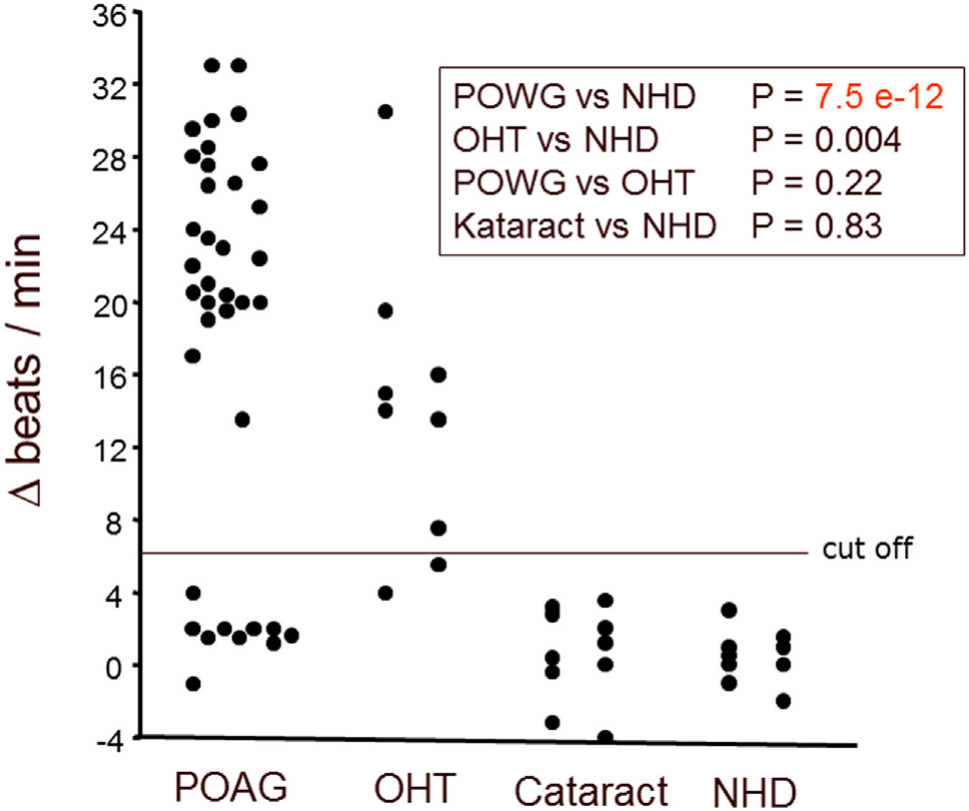
Antibodies stimulating rat cardiomyocytes in sera of patients with POAG and OHT. Immunoglobulin-enriched serum fractions were added to cardiomyocyte cultures in a 1:20 dilution and incubated for 60 min at 37°C. The data are displayed as the changes of the beats/min. Each point represents 6–10 separate single-cell check points in a culture flask. Repeated measurements of the effects with different cell cultures prepared on different days resulted in almost identical data. The differences between patients with POAG or OHT versus healthy controls were highly significant. Primary open-angle glaucoma (POAG, *n* = 37); ocular hypertension (OHT, *n* = 9); cataract (*n* = 10); healthy donors (NHD, *n* = 10).

Immunoglobulin-enriched fractions of sera of patients with POAG, OHT, cataract, and of healthy donors (NHD) were analyzed for AAbs against GPCRs. We calculated the cutoff as mean of the beating rate in normals added to three times of the SD. As shown in Figure 1, the beat rate of cardiomyocytes was increased by the immunoglobulin fractions in POAG, OHT, yet not in cataract and NHD. 73% of the POAG patients and 78% of the OHT patients showed increased beat rates of the cardiomyocytes, whereas 0% of the cataract and NHD yielded an increased beat rate. This indicates the presence of agonistic AAb in patients with POAG and OHT. To identify the specific target(s) and IgG fraction of these AAb receptor-specific analyses were performed.

### Immunoglobulins from POAG and OHT *Patients Interact* with the β2AR

**Figure 2:**
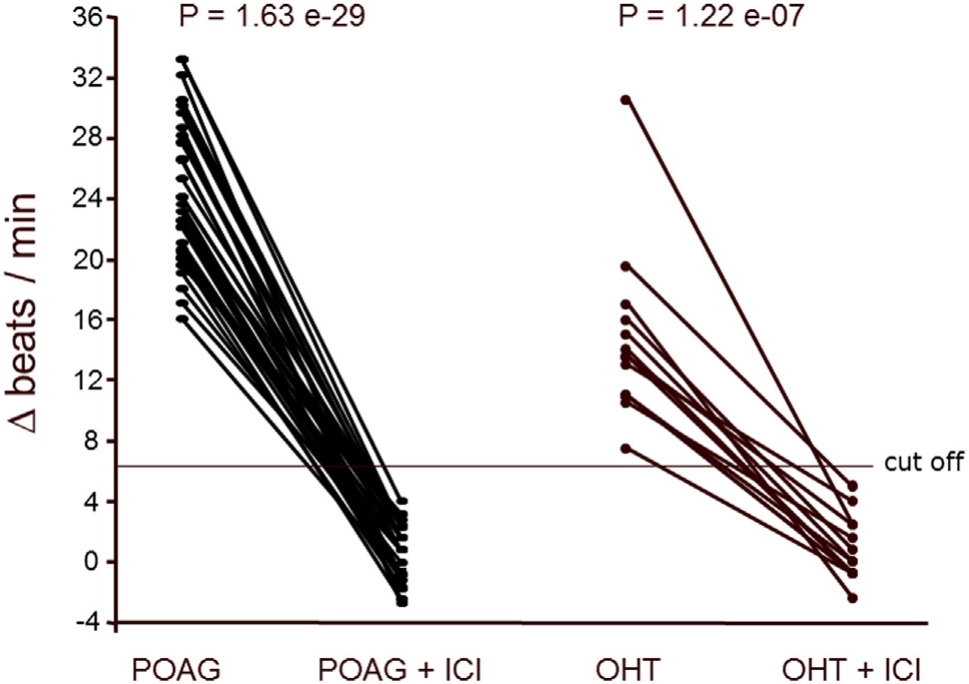
β2-adrenergic receptor (β2AR) agonistic autoantibodies (AAbs) in patients with primary open-angle glaucoma (POAG) or ocular hypertension (OHT). The neonatal cardiomyocytes were incubated with the immunoglobulin fractions from POAG (*n* = 33) and OHT (*n* = 14) patients as described in Figure 1. The agonistic effect of the AAbs was blocked by the β2AR antagonist ICI118551 (ICI; 0.1 μM). ICI (0.1 μM) did not affect the basal beating rate of the cardiomyocytes.

The positive chronotropic responses of the AAb from patients with POAG and OHT were analyzed for their receptor specificity using respective antagonists. The action of AAb on the cardiomyocytes was blunted by ICI118.551 and the β1/β2 adrenergic antagonist propranolol (Figure 2), but not by bisoprolol (β1AR antagonist) or losartan (AT1 antagonist; not shown). The β2AR-specific agonist clenbuterol enhanced the cardiomyocyte beat rate of all samples, indicating functionality of the detection system (not shown).

**Figure 3:**
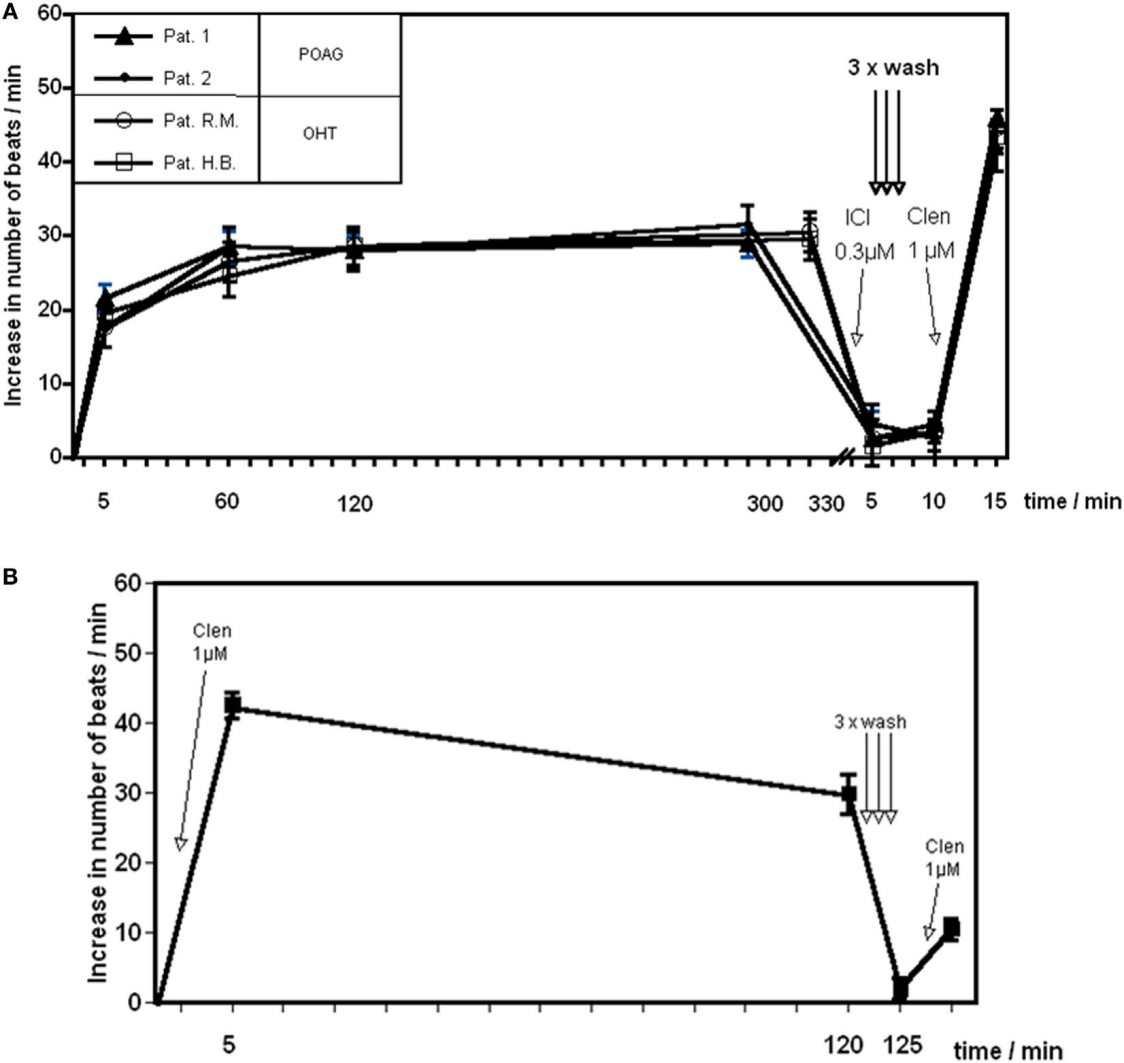
Time response curve of β2 adrenergic agonists. Effect of **(A)** the agonistic autoantibody (AAb) prepared from patients with primary open-angle glaucoma (POAG) or ocular hypertension (OHT) on spontaneously beating rat cardiomyocytes and **(B)** clenbuterol (Clen). Clenbuterol induced a desensitization of the β adrenergic response within 2 h. After 2 h, clenbuterol was washed out and restimulation with clenbuterol resulted in approximately 30% of the initial stimulation **(B)**. β2-adrenergic receptor AAb showed no desensitization for at least 5 h **(A)**. Before washing, the antibodies were removed from the receptors by ICI118551. After washing, the cells react to a clenbuterol stimulation with a maximal response (*n* = 10 for each point of measurement).

Reportedly, agonistic AAb against the β1 adrenoceptor or monoclonal antibodies against the second extracellular loop of the β2 adrenoceptor did not desensitize the corresponding receptors within 4 to 6 h (49). Therefore, we analyzed the long term effect of the β2 AAb prepared from patients with POAG. The AAb did not induce desensitization of the β2AR within 5 h. ICI118.551 normalized the beating rate of the cardiomyocytes. A successive washing procedure removed ICI118.551 and the AAbs. When the washed cells were stimulated with the β2 agonist clenbuterol, a maximal response to this agonist was to be observed (Figure 3A). On the other hand, clenbuterol desensitized the receptor mediated signal cascade and decreased the beating rate of the cardiomyocytes. After washing out the agonist and a further stimulation with clenbuterol, we observed an increase of the beating rate by only 30% of the response obtained in the first stimulation with clenbuterol. We conclude that, in contrast to clenbuterol (Figure 3B), AAb did not desensitized the β2AR mediated signal cascade.

### The β2 AAb Recognize Two Peptide Epitopes of the Second Extracellular Loop II of the β2AR

**Figure 4:**
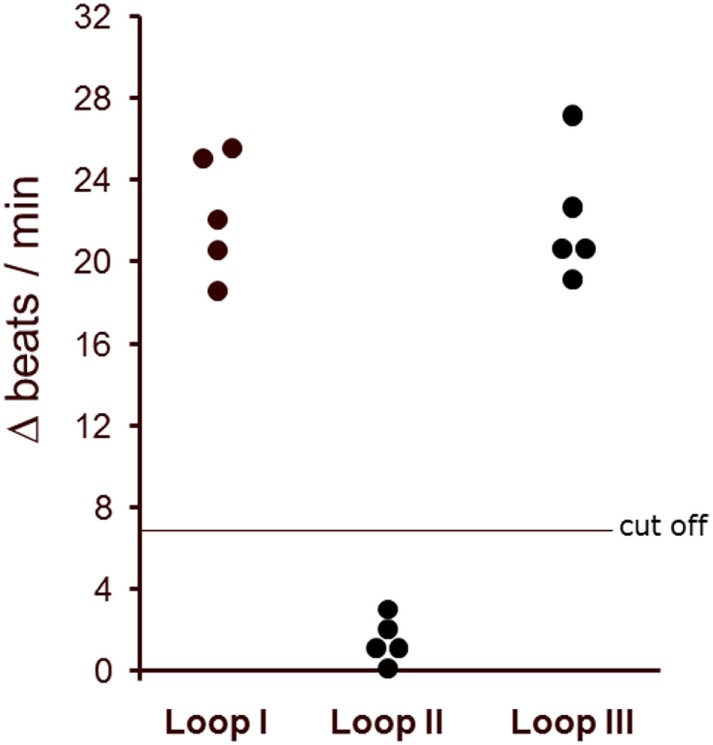
The agonistic anti-β2AR autoantibodies (AAbs) of patients with primary open-angle glaucoma (POAG) recognized the second extracellular loop of the β2-adrenergic receptor. The antibody preparations from patients with POAG (*n* = 5) were pretreated with loop-specific peptides corresponding to the extracellular loops I–III. The antibody–peptide complexes were added to the cardiomyocytes to measure remaining agonistic capabilities. The final AAb dilution was 1:40. The data are presented as the increase in beats/min versus baseline beating rates.

The binding of the AAb to distinct extracellular loops of β2AR was characterized in the presence of loop-specific synthetic peptides. As presented in Figure 4 only the loop II peptide neutralized the agonistic activity. This indicates that the second extracellular loop of β2AR is the target of the AAb in the sera of patients with glaucoma.

**Figure 5:**
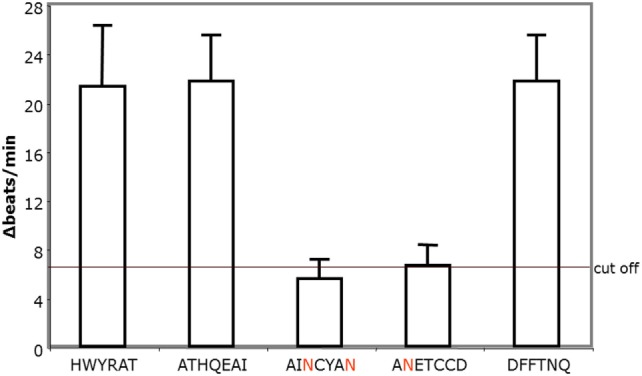
The agonistic anti-β2AR autoantibodies (AAbs) of patients with primary open-angle glaucoma (POAG) recognize the epitopes AINCYAN and ANETCCD of loop II. The procedure to detect epitope-specific responses of immunoglobulins isolated from POAG patients is similar to the one used in the studies for loop analyses. The final AAb dilution was 1:40. Data are displayed as individual measurements of five independent patients with POAG.

To identify the epitopes of the AAbs, we pretreated the AAb with short overlapping peptides corresponding to the second extracellular loop of the β2AR. For these studies five synthetic oligopeptides were prepared covering the loop II amino acid 172–197. Only the loop II-borne peptides AINCYAN (181–187) and ANETCCD (186–192) abolished the agonistic activity of the AAb from five representative patients with POAG (Figure 5) and, thus, represent the dominant epitope of the β2AR.

### Affinity Purified AAb of POAG and OHT Patients Induce Agonistic Effects in a Dose-Dependent Manner

Affinity purified AAb against the β2AR exert a dose-dependent positive chronotropic effect on cultured spontaneously beating rat cardiomyocytes. The maximal response for the POAG and OHT was observed at a dilution of the antibodies of 1:200 and 1:100, respectively.

### Surface Plasmon Resonance Analysis Confirmed the Receptor Specificity of the Agonistic AAb

Surface plasmon resonance allows to determine direct physico-chemical interactions between macromolecules. Analyzing the IgG fractions of sera from patients with glaucoma we observed a significant binding activity to a loop II peptide from the β2AR (Table 6). We subjected the values to a statistical four-field analysis. The Fisher-test revealed a significance of *p* = 0.01282, a relative risk (CI) of 0.37721 (0.37093–0.84993), a sensitivity of 0.35 and a specificity of 0.0066. Therefore, the AAb from the sera of patients with glaucoma display a higher affinity for the loop II of the β2AR than those of controls.

**Table 6:** Surface plasmon resonance analysis of binding of IgG fractions to the biotinylated H19C peptide (biotinyl-LC-HWYRATHQEAINCYANETC) derived from loop II of the β2AR.

Proband	Initial association phase slope on H19C peptide
1	0.441
2	0.0579
3	−2.98E−03
4	0.102
5	0.0439
6	0.0572
7	−0.0544
8	0.333
9	−0.0224
10	0.0955
11	0.102
12	0.176
13	0.413
14	0.326
15	0.11
16	0.396
17	0.115
18	0.356

### The *Agonistic* β2AR AAb in the *Sera* of *Patients* with POAG Are of the IgG3 *Isotype*

**Figure 6:**
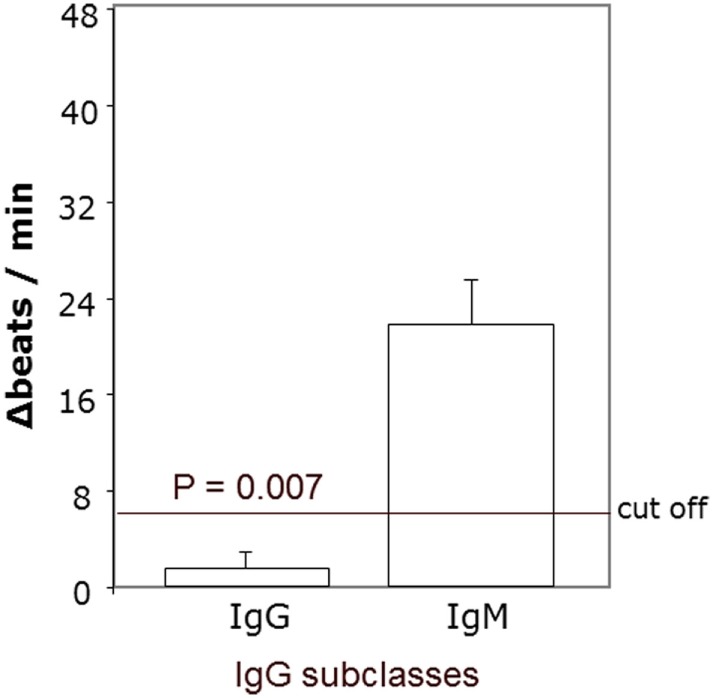
Agonistic anti-β2AR IgG autoantibodies in patients with primary open-angle glaucoma. The neutralization of IgG from the immunoglobulin-enriched fraction lead to the loss of the stimulatory potential. Antibodies against IgM had no effect. The β2 receptor agonist clenbuterol served as positive control (not shown).

**Figure 7:**
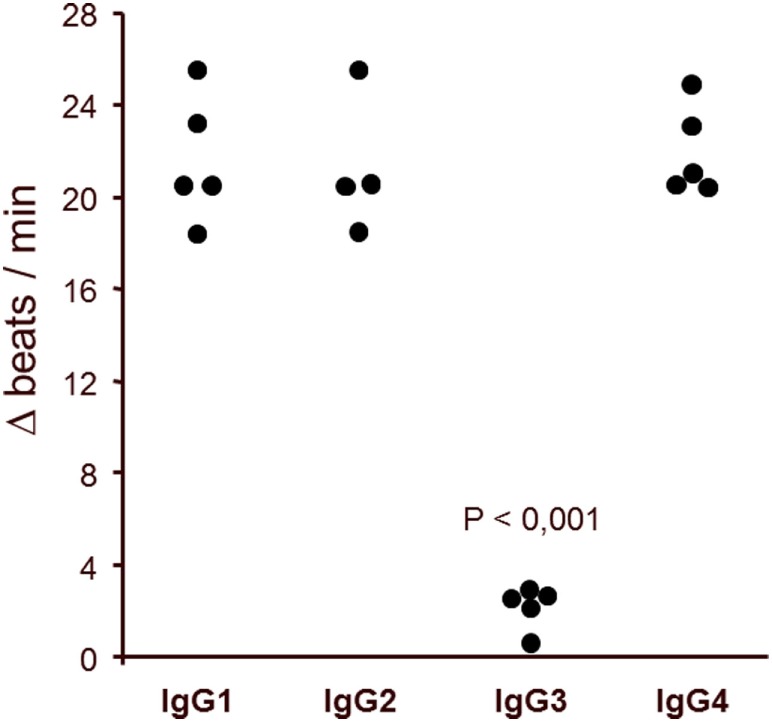
IgG3 subclass of the agonistic anti-β2-adrenergic receptor autoantibodies in patients with primary open-angle glaucoma. The neutralization of IgG3 from the immunoglobulin-enriched fraction leads to the loss of the stimulatory potential. Antibodies against IgG1, 2, and 4 had no effect. The β2 receptor agonist clenbuterol served as positive control (not shown).

To define the nature of the molecules in immunoglobulin-enriched fractions, which increased beating rates of cardiomyocytes, we used antibodies against immunoglobulins IgG and IgM (Figure 6). Only anti-IgG significantly prevented the agonistic activity. We next analyzed the IgG subclass of the agonist-like β2AR AAb with antibodies specifically depleting certain IgG subclasses (Figure 7). Only IgG3-precipitating antibodies eliminated the agonistic activity. Therefore, most of the agonistic AAb are of the IgG3 isotype.

### Clinical Parameters in Correlation to AAbs

We analyzed the clinical parameters of the patients with respect to treatment for glaucoma, IOP, and general diseases including cardiovascular disease and diabetes (Tables 3 and 4). We could not detect any correlation of these parameters between AAb positive POAG and OHT patients with NHD or AAb negative patients (data not shown).

### Pilot Proof-of-Principal Study on IA for Patients with POAG

**Figure 8:**
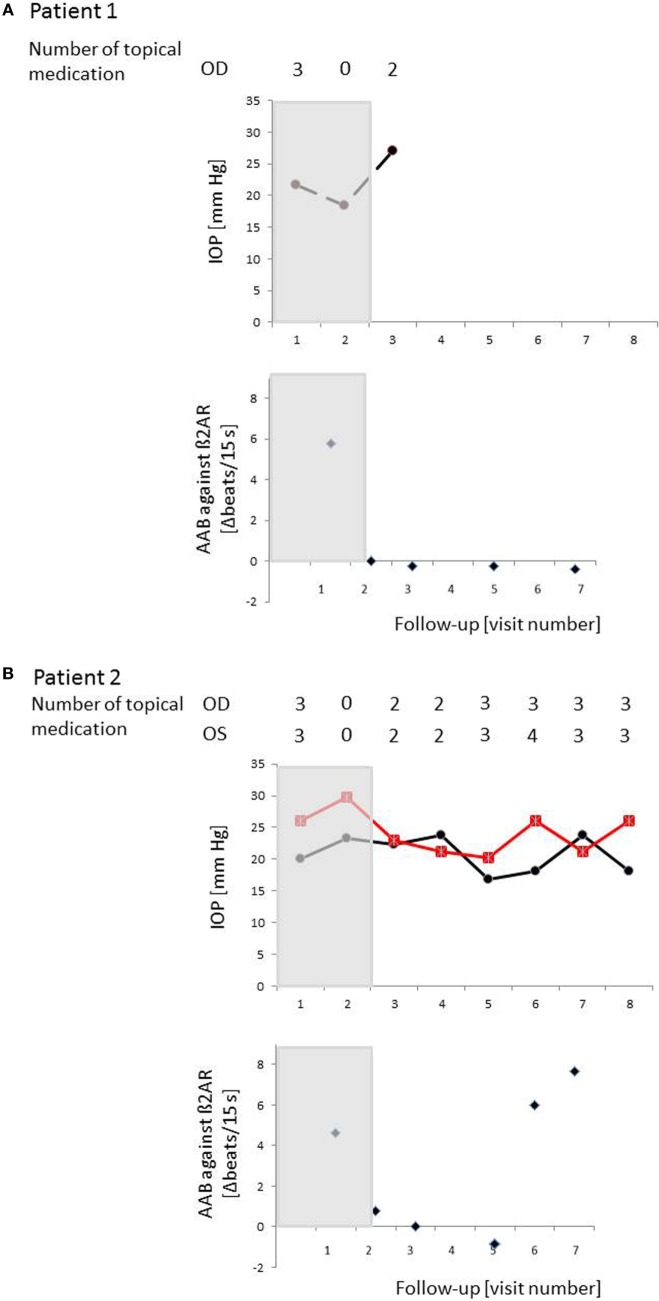

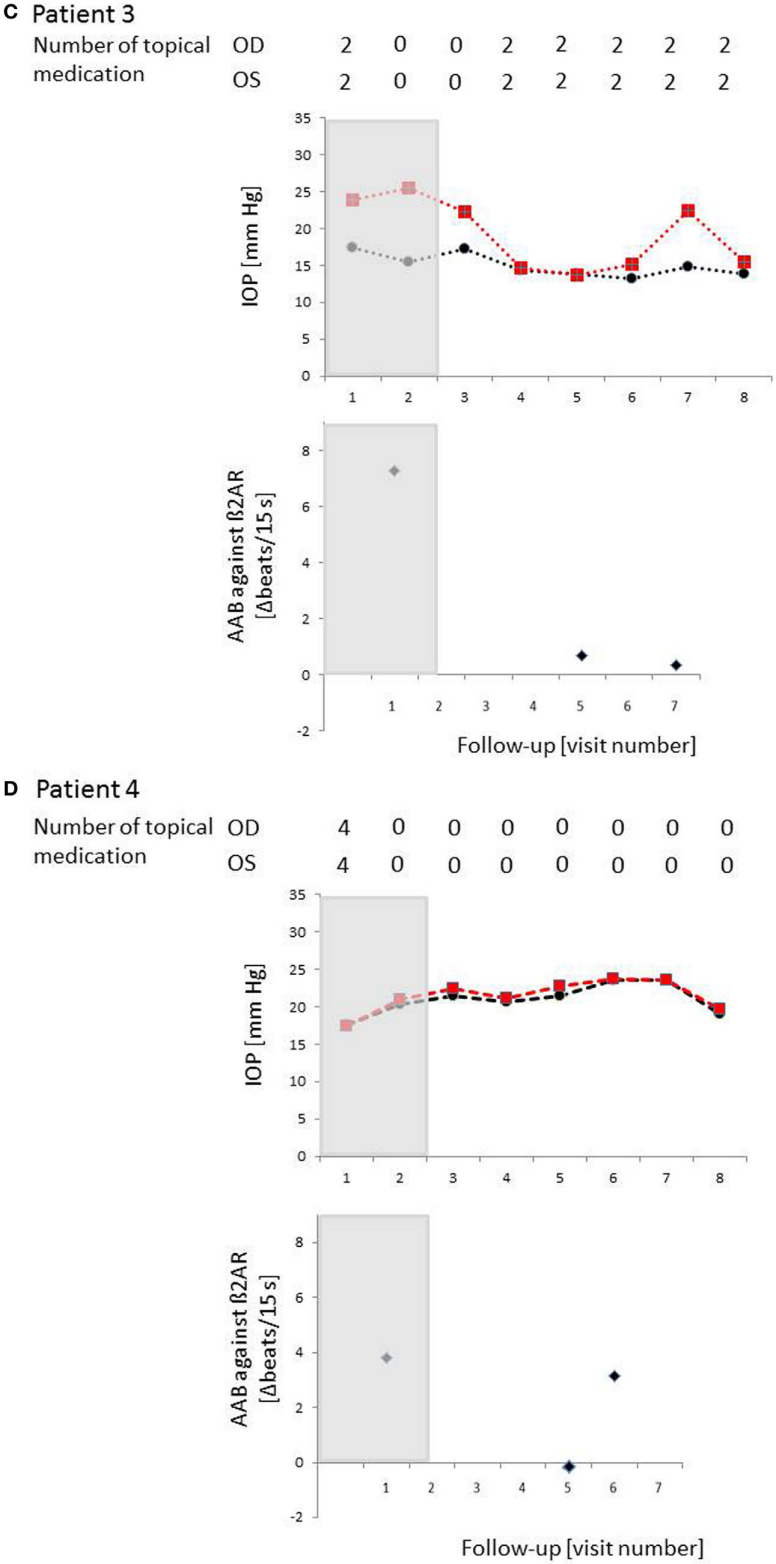
Time course of IOP, levels of autoantibodies (AAbs) and number of local antiglaucomatous eye drops during immunoadsorption (IA) for the patient with primary open-angle glaucoma (POAG). Patient 1/2/3/4 (A)/(B)/(C)/(D): a decrease in IOP under reduced number of local antiglaucomatous therapy was to be observed in all patients, going along with a decrease of the AAb. Intraocular pressure (IOP) increased, when AAbs relapsed after the IA; OD, right eye (black); OS, left eye (red); grayish area, data before IA; visit 1, IOP graph: less than 3 months before immunoadsorption under maximal combination therapy, AAB against β2AR graphs: before immunoadsorption; visit 2 – IOP graph: two days before immunoadsorption with system medications (acetazolamide), yet without topical medications, AAB against β2AR graphs: at the end of immunoadsorption; IOP/AAB against β2AR graphs: visit 3–2 weeks after immunoadsorption; visit 4–1 month after immunoadsorption; visit 5–2 months after immunoadsorption; visit 6–4 months after immunoadsorption; visit 7–6 months after immunoadsorption; visit 8–12 months after immunoadsorption.

The IA was well tolerated in all patients—no adverse events were to be observed. Specifically, no change in visual acuity, visual field, and intraocular flare measured by laser flare cell meter was detected (not shown). The levels of the AAb before and after IA are shown in Table 7. The IOP course is presented in Table 8 for each patient. Time course of IOP and levels of AAbs during IA are shown in Figure 8.

**Table 7:** AABs, IgG (mg/dl), and IgG3 (mg/dl) before and after immunoadsorption (IA).

		Before IA	End of IA	After IA			
				2 weeks	2 months	4 months	6 months
1	AAB	5.8	0	−0.25	−0.25		−0.38
	IgG	1,190	134	469	859		1,000
	IgG3	51.8	16.3	32.5	38.1		42.2

Date of examination		(25.09)	(29.09.)	(06.10)	(09.11)		(16.05 of following year)
2	AAB	4.63	0.75	0	−0.88	6.0	7.67
	IgG	1,500	122			1520	1,410
	IgG3	44.8	15.1			37.1	47.6

Date of examination		(13.11)	(17.11)			(07.02 of following year)	(22.06 of following year)
3	AAB	7.25				0.67	0.33
	IgG	1,370	178	574	914	1240	
	IgG3	49.1	17.9	31.8	59.1		

Date of examination		(07.05)	(11.05)	(25.05)	(20.06)	(21.08)	
4	AAB	3.83			−0.17	3.17	
	IgG	1,150	92.0	773	1030		1,020
	IgG3	54.2	15.9	61.4			91.3
Date of examination		(04.08.)	(08.08)	(25.08)	(14.10)		(17.02 of following year)

**Table 8:** Intraocular pressure (IOP) before and after immunoadsorption (IA)—Visit 0: less than 3 months before IA under maximal combination therapy.

Patient		Before IA				After IA											
		Visit 0		Visit 1		2 weeks		1 month		2 months		4 months		6 months		12 months	
		IOP	Med	IOP	Med	IOP	Med	IOP	Med	IOP	Med	IOP	Med	IOP	Med	IOP	Med
1	OD	21.7 ± 4.0 (17–26, 9)	3	18.4 ± 2.8 (13–21, 8)	0	27.0 ± 6.5 (20–37, 17)	2	TE									

2	OD	20.0 ± 2.0 (17–23, 6)	3	23.3 ± 3.7 (17–33, 16)	0	22.4 ± 3.9 (17–29, 12)	2	23.8 ± 4.5 (19–30, 11)	2	16.8 ± 1.9(15–20, 5)	3	18.2 ± 2.3 (15–22, 7)	3	23.8 ± 4.5 (19–30, 11)	3	18.2 ± 2.3 (15–22, 7)	3
	
	OS	26.0 ± 4.5 (21–33, 12)	3	29.7 ± 4.6 (22–40, 18)	0	23.0 ± 4.4 (19–30, 11)	2	21.2 ± 3.8 (16–26, 10)	2	20.2 ± 2.3 (18–24, 6)	3	26.0 ± 3.0 (22–31, 9)	3	21.2 ± 3.8 (16–26, 10)	3	26.0 ± 3.0 (22–31, 9)	3

3	OD	17.4 ± 3.7 (12–24, 12)	2	15.5 ± 5.2 (10–23, 13)	0	17.2 ± 6.9 (12–31, 19)	0	14.3 ± 3.5 (10–19, 9)	2	13.8 ± 0.4 (12–14, 1)	2	13.2 ± 1.6 (12–16, 4)	2	14.8 ± 3.4 (12–19, 7)	2	13.8 ± 2.4 (12–16, 4)	2
	
	OS	23.9 ± 7.9 (16–40, 24)	2	25.5 ± 8.4 (16–42, 26)	0	22.3 ± 14.6 (14–52, 38)	0	14.7 ± 3.6 (10–19, 9)	2	13.7 ± 0.8 (12–16, 2)	2	15.2 ± 1.8 (12–17, 5)	2	22.5 ± 6.8 (16–32, 16)	2	15.5 ± 2.4 (12–19, 7)	2

4	OD	17.6 ± 3.3 (11–25, 14)	4	20.4 ± 2.8 (18–24, 6)	0	21.5 ± 5.4 (14–30, 16)	0	20.7 ± 5.3 (15–26, 11)	0	21.5 ± 5.4 (14–29, 15)	0	23.5 ± 4.6 (17–28, 11)	0	23.5 ± 4.6 (17–28, 11)	0	19 ± 1.8 (17–22, 5)	0
	
	OS	17.4 ± 2.9 (12–23, 11)	4	21.0 ± 2.5 (18–24, 6)	0	22.4 ± 4.3 (17–30, 13)	0	21.2 ± 4.7 (16–26, 10)	0	22.8 ± 4.5 (16–29, 13)	0	23.7 ± 4.1 (20–28, 8)	0	23.5 ± 4.0 (20–28, 8)	0	19.7 ± 4.0 (15–26, 11)	0

*Visit 1: two days before IA with systemic medications (acetazolamide), yet without topical medications. Mean ± SD (minimum–maximum, range) of 24 h IOP curve are presented*.

The individual IOP course of each patient was as follows.

#### Patient 1 (Male, 71 Years Old)

Mean IOP before immune adsorption of the first patient showed a reduction only during the first days of IA (Figure 8A. The IOP dropped from 15.6 ± 1.8 mmHg to 15.1 ± 03 mmHg (3.2%) during the first day and raised to 23.9 ± 1.9 mmHg during the last day of IA. Because of the advanced stage of glaucoma, trabeculectomy was performed 4 weeks after IA.

#### Patient 2 (Female, 52 Years Old)

In the second patient largest reduction of IOP was found 2 months after IA (visit 4) (Figure 8B). IOP dropped about 27.8% at the right eye (OD) and 32.1% at the left eye (OS) if compared to IOP at visit 1. At visit 7 (12 months after IA) mean IOP was still decreased by about 21.9% at the right eye and 12.5% at the left eye in comparison to visit 1.

#### Patient 3 (Female, 56 Years Old)

Largest drop of IOP was found in the third patient (Figure 8C). At visit 4, the reduction of IOP was 11% for the right eye and 46.3% for the left eye with additional two topical medications. Without topical medications, IOP reduction was found to be 12.5% at the left eye, yet IOP increase of about 11% at the right eye (visit 2, 2 weeks after IA) compared to IOP at visit 1.

#### Patient 4 (Male, 57 Years Old)

The fourth patient showed the lowest IOP measurement (without topical medication) at the end of the observation period (without medication visit 7) (Figure 8D). One month after IA, IOP was equal to IOP before IA (without medication, visit 1) with an increase of mean IOP up to 23.5 ± 4.6 mmHg at the right eye and 23.5 ± 4.0 mmHg at the left eye (6 months after IA, visit 6). In comparison to visit 0 (three medications), no topical medication was necessary at the end of the observation period.

Taking together, in all patients, AAb and other IgGs were washed out almost completely by IA, resulting in autoantibody concentrations below the detection limit at the end of treatment. In two patients (1 and 3) AAb remained undetectable during the 6 months follow-up period. The two other patients showed detectable AAb-levels 4 months after IA. The reduction of total IgG and IgG3 levels ranged from 88 to 95 and from 64 to 80%, respectively. Four weeks after IA total IgG and IgG3 concentrations reached normal values.

## Discussion

In the present study, we describe formerly unknown circulating AAb in patients with glaucoma (50). We screened patient sera for antibodies that activate a GPCR expressed by cardiomyocytes and detected AAb against the β2AR employing specific inhibitory peptides. These agonistic AAb induced a dose-dependent stimulation and their effects were blocked by the selective antagonist ICI118.551, and neutralized by peptides corresponding to the second extracellular loop of the β2AR (AS 181–192).

Autoantibody can be detected by several methods: (1) enzyme-linked immunosorbent assay detects the patient’s antigen bound AAb by a specific (to human IgG) animal sourced and enzyme (peroxidase or alkalic phosphatase) labeled antibody; (2) fluorescence (microscopy) detects AAb as (1), but the detection of the AAb is fluorescence labeled (e.g., fluorescein isothiocyanate, FITC); (3) radioimmunoassay detects AAb as (1), however, the detection of the AAb is radio labeled (e.g., iodine); (4) bioassay (with spontaneous beating neonatal rat cardiomyocytes) measures the increase in the beating rate, induced by added IgG (fraction from human serum) due to the binding of AAb to the corresponding receptor (here: β2-AR). The detection methods (1)–(4) measure the AAb indirectly, however method (4) additionally directly. As the detected signal extent of the bioassay is proportional to the AAb bound to the surface fixed target, we decided to choose the cardiomyocyte bioassay for the detection of the AAb. Next to the detection of the AAbs, this method enables functional analyses.

The AAb found in glaucoma patients are of the IgG3 subclass. The antibodies that trigger effector functions, and that are most likely to be involved in immunoregulatory activity, are IgG3 and IgG1 (51). In addition, it is known that IgG3 is involved in antibody-dependent cellular cytotoxicity (52). Complement activation is most effective with IgG3 and, to a lesser extent, with IgG1, IgG2, and IgG4. Usually, complement binding and activation leads to destruction of the target structure. The plasma concentration of IgG3 is low, and its half-life is shorter than that of any of the other IgG-subclasses. Further investigations are necessary to elucidate whether complement activation and cell death are involved in the pathogenesis of glaucomatous diseases.

Recent studies support the hypothesis that glaucoma-associated neurodegenerative processes are partially caused by immune-mediated mechanisms (53–56). These studies report complex AAb repertoires in patients with glaucoma which were directed against retinal and optical nerve antigens. Such autoantibody reactivities were shown in optical nerve extracts from bovine eyes. The later findings are of interest in respect to ocular pressure-dependent and independent autoimmune responses. With regard to AAb-mediated immunoreactions, these pathomechanisms seem to be driven by inflammation.

The β2AR is one of the best investigated GPCR. The modulation of cardiac contractility is also mediated by β2AR, and its activation might have chronic effects on cellular metabolism, on cell growth, or on excitability (57). This receptor seems to be involved in allergic asthma, several cardiomyopathies, such as Chagas’ disease, and cardiac electrical disturbances (58–60). However, whereas the AAb in the case of Chagas disease were functionally active in cardiomyocytes, β2-AAb from asthmatic patients react in the opposite way. Respective loop analyses explained these findings; in the Chagas-related cardiac disorders the loop II was identified to be the target of AAbs, whereas in the asthmatic disease the loop III was recognized. Thus, it was concluded that the various extracellular loops of the β-AR are functionally linked to distinct antibody-mediated responses.

Several years ago, the effect of monoclonal antibodies directed against the second extracellular loop of the beta2 adrenoceptor was investigated. Interestingly, similar results were detected as seen with the AAbs prepared from the sera of glaucoma patients (61). The agonist-like effect of this mAB (6H8) was blocked by the specific β2-adrenergic antagonist ICI118.551 and a peptide HWYRATHQEAINCYANETC corresponding to the second extracellular loop of the β2-adrenoceptor. The β1-adrenoceptor antagonist bisoprolol was without influence. The AAb were directed against loop II and recognized the overlapping epitopes AINCYAN and ANETCCD. Considering that the peptide corresponding to the second extracellular loop and the short overlapping peptides cannot form the correct conformation of the extracellular loop, the anti-β2 receptor antibodies were pretreated with the peptides corresponding to the first, second or third extracellular loop for 1 h. Under these conditions, only the peptide that corresponds to the second extracellular loop was able to neutralize the AAb activity. The peptides of the first and third extracellular loops were without effect. Similar results we observed also for the short overlapping peptides with an amino acid sequence of 5–6 amino acids. In these experiments, the sequence (AINCYANETCCD) was able to neutralize the AAb action. Therefore, it is assumed that this sequence represents the epitope of the AAb on the second extracellular loop. Plasmon resonance and affinity purification confirmed the direct interaction of the AAb with loop II of the β2AR. The agonistic β2AR antibodies described here are good mechanistic candidates that might induce and maintain an elevated IOP as a critical step in the development of glaucomatous diseases.

In this study, we present first *in vivo* data of four patients with POAG that underwent IA in a pilot proof-of-principal study: glaucoma therapy is considered to be effective when IOP reduction is 20% from wash out baseline (62). Even not all patients in a study group must show a substantial IOP decrease of 20%, in cases when representative parts of the patients display even more than 20% IOP reduction. Thus, an IOP decrease could be observed in 3 of 4 patients after IA: patients 2 and 3 showed an IOP decrease of 27.8% (OD)/32.1% (OS) and 11% (OD)/46.3% (OS) using equal number of topical antiglaucomatous eye drops. An IOP decrease of 2.9% (OD) and 6.2% (OS) could be observed 12 months after IA in patient 4. As it is known that each single millimeter of mercury increases glaucoma progression rate about 12–13% (18) even the IOP decrease in this patient is important and enables winning sighted lifetime. The IOP of patient 4 is considered to be even lower after IA as the number of local antiglaucomatous eye drops decreased from 4 to 0, thus, measured IOP values under no local therapy can be seen as a lowered IOP after IA with even higher IOP under quadruple therapy before IA. Summarizing, immunoadsorption seemed to lower IOP or even number of antiglaucomatous therapy. Possibly, the decrease of IOP could even be larger, if β2AR AAbs are blocked specifically. Because IA is a non-specific method for adsorbing AAbs and other IgGs, several other AAb were removed in addition to the agonistic β2AR AAb. As known for other autoimmune diseases, AAb relapse. This effect was also seen in our patients. Detection of β2AR AAbs went along with an IOP increase (patients 2 and 4). The delay in the decrease of IOP might be possibly due to a re-sensitization of the β2AR in the absence of stimulating AAbs. In addition, the therapy was well tolerated and no adverse effects were observed.

The most important target of glaucoma treatment is elevated IOP. A large body of evidence has established the importance of the reduction of the IOP in the medical management of glaucomatous diseases (13–17, 63). The pathogenic damage of the optic nerve in POAG is not well understood. It is supposed to be associated with increased IOP, glutamate toxicity (64), interrupted transport of neutrophins (8), apoptosis (4, 65), extracellular matrix changes (5, 6), and hypoxia due to ocular and systemic vascular dysregulation (66).

The aqueous humor dynamic plays the major role in the regulation of IOP. Four factors maintain the steady-state IOP of the healthy eye. These are flow of aqueous humor, resistance to outflow, episcleral venous pressure (67), and uveoscleral outflow (68). The IOP reflects the balance between in- and outflow of aqueous humor. A major strategy in medical treatment of glaucoma is the reduction of inflow and, thereby, normalization of IOP. Understanding the mechanisms and regulation of inflow is, thus, of undoubted clinical relevance. Several mechanisms underlying increased inflow have been identified, however, the integration and regulation of these mechanisms is still elusive (69). The balance of in- and outflow of aqueous humor is modulated by the local β2AR activity and may be chronically influenced by agonistic AAbs.

Usually, night-time aqueous flows are approximately half of those observed at daytime in active humans (70–72). The ability of aqueous flow stimulation by epinephrine during night-time shows a low basal β-adrenergic stimulation during the night (23). Patients with POAG form in average 15% more aqueous humor/24 h than control subjects of the same age. This is due to a significantly higher aqueous flow in the night in comparison to control subjects (73). Thus, a β-adrenergic overdrive precludes the downregulation of the aqueous flow during the night. This observation is underlined by the lack of receptor desensitization by agonistic AAb displayed in Figure 3. Usually, β2AR agonists such as clenbuterol desensitize the receptor within 1–2 h (Figure 3B). By contrast, the β2AR AAb do not desensitize the β2AR *in vitro* for at least 5 h. This chronic β2-adrenergic stimulation may explain the higher aqueous flow and the elevation of the IOP in patients with POAG. Alteration of the circadian rhythm of IOP in POAG can be explained by the presence of AAb. The lack of tachyphylaxia was also observed for agonistic antibodies against anti-β1AR-, anti-α1AR-, anti-AT1-, and anti-muscarinic M2 receptor in several cardiovascular diseases (74, 75).

The AAb might also play a modulatory role in the aqueous outflow. Putney et al. (76) found an increase of cell volume of trabecular meshwork cells *via* Na^+^-K^+^-Cl^−^ cotransporters in glaucoma leading to a reduced outflow. As β-adrenergic stimulation results in an activation of Na^+^-K^+^-Cl^−^ cotransporters, agonistic AAb might also contribute to reduced outflow facility.

An “organ specificity” for the AAb against the β2AR can be assumed as patients of glaucoma suffered not of other diseases, which can be caused by these AAb (e.g., allergic asthma). It can be hypothesized that the chemical surrounding has to be changed before the autoantibodies can act specifically. Previous data showed that the βAR is not accessible for the AAb under adequate oxygen conditions. However, the βAR seemed to be unmasked, if ischemia occurred (77, 78). This could be a potential reason as glaucoma is hypothesized to be a disorder with local ischemia (79).

The data presented here offer another insight into eye disorders associated with high IOP. The newly identified AAb against the β2AR were exclusively found in the sera of patients with POAG and OHT but neither in those with cataract, nor in controls. Based on these observations, we suggest that these AAbs are not only immunological markers with a high incidence in POAG and OHT, but might also be pathomechanistically relevant β2AR activating molecules. The common, broadly successful therapy with β2AR antagonists further supports the idea that AAb-mediated mechanisms might be of pathogenic importance. In addition to their effect on IOP, the AAb might also have a direct effect on retinal ganglion cells as β-adrenoreceptor antagonists showed neuroprotective properties (80).

## Conclusion

In patients with POAG and OHT, however, not in controls, agonistic AAb directed to β2AR were detected. These AAb interacted with the second extracellular loop of β2AR (peptides 181–187 and 186–192), thus functionally different links to distinct antibody-mediated responses are assumed. Data of our clinical pilot proof-of-principal study might suggest that a reduction of total IgG, including AABs against β2AR (IgG3 isotype) by IA, transiently decreases IOP. These findings might indicate a possible role of these AAb in the dynamics of aqueous humor and might support a contribution of adaptive autoimmunity in the etiopathogenesis of POAG and OHT. A specific blocking of AABs against β2AR might be of interest for further studies.

## Ethics Statement

The study followed the tenets of the declaration of Helsinki for research. Written informed consent to use serum samples for research purposes was obtained from each participants of the study. The institutional review board of the University Hospital Erlangen approved the protocols (3483; ClinicalTrials.gov Identifier NCT00494923).

## Author Contributions

RK, MH, and GW had the idea and planned the study; GW, QF, and SB performed the laboratory work; AJ, JR, and RV planned and performed the clinical trial; HK did the synthetic work; JH did laboratory work; AS and BH performed data acquisition and statistical analysis; RR was involved in the analysis and revised the manuscript, FH was involved in the analysis, BH was responsible for the draft of the manuscript. US-S interpreted results and edited the manuscript. All authors have no financial interest in any topic regarding the present study and approved the final version of the manuscript.

## Conflict of Interest Statement

(1) Patent: EP 1832600 A1 (2) RK is working for Fresenius Medical Care GmbH. The reviewer RK declared a shared affiliation, with no collaboration, with one of the authors, HK, to the handling editor.
